# Merkel Cell Polyoma Viral Load and Intratumoral CD8+ Lymphocyte Infiltration Predict Overall Survival in Patients With Merkel Cell Carcinoma

**DOI:** 10.3389/fonc.2019.00020

**Published:** 2019-01-24

**Authors:** Jens von der Grün, Ria Winkelmann, Markus Meissner, Ulrike Wieland, Steffi Silling, Daniel Martin, Emmanouil Fokas, Claus Rödel, Franz Rödel, Panagiotis Balermpas

**Affiliations:** ^1^Department of Radiotherapy and Oncology, University of Frankfurt, Frankfurt, Germany; ^2^Dr. Senckenberg Institute of Pathology, University of Frankfurt, Frankfurt, Germany; ^3^Department of Dermatology, University of Frankfurt, Frankfurt, Germany; ^4^Institute of Virology, National Reference Center for Papilloma and Polyomaviruses, University of Cologne, Cologne, Germany; ^5^German Cancer Research Center (DKFZ), Heidelberg, Germany; ^6^German Cancer Consortium (DKTK) Partner Site Frankfurt, Frankfurt, Germany; ^7^Department of Radiation Oncology, University Hospital Zürich, Zurich, Switzerland

**Keywords:** merkel cell carcinoma, polyomavirus (MCPyV), CD8+ tumor infiltrating lymphocytes, PD-L1, radioimmunotherpay

## Abstract

**Introduction:** Merkel cell carcinoma (MCC) is linked to the presence of clonally integrated Merkel cell polyomavirus (MCPyV) in up to 80% of the cases. The aim of the study was to determine the prognostic value of baseline MCPyV viral load and lymphocytic infiltration.

**Methods:** MCPyV DNA prevalence, integration status and viral load were determined by specific quantitative real-time PCR in surgical specimens obtained from 49 patients with MCC treated with (*n* = 22, 45%) or without postoperative radiotherapy (RT). CD8+ tumor infiltrating lymphocytes (TILs) and programmed death ligand 1 (PD-L1) status were assessed using immunohistochemistry. MCPyV characteristics and immune marker expression were correlated with clinicopathological factors and overall survival (OS).

**Results:** Median age at diagnosis was 74 (range, 42–100); 51% of the patients were female. One-, three, and five-year OS rates were 83.8, 58.6, and 47.1%, respectively. A positive MCPyV status was associated with female gender (*p* = 0.042). Tumor localization (head/arms vs. trunk) positively correlated with PD-L1 status (*p* = 0.011) and combined CD8/PD-L1 expression (*p* = 0.038). Overall CD8+ infiltration was inversely associated with N-stage (*p* = 0.048). Stromal TILs correlated significantly with both PD-L1 expression (*p* = 0.010) and N-stage (*p* = 0.037). A high viral load (>median) was significantly associated with worse OS (*p* = 0.029) and high intratumoral CD8+ infiltration with improved OS for the entire cohort (*p* = 0.045).

**Conclusion:** These data provide important insight on the role of MCPy DNA viral load and TILs in the context of PD-L1 in patients with Merkel cell carcinoma. Future clinical studies should aim to explore the effect of PD-1/PD-L1 immune-checkpoint inhibitors in combination with existing radiotherapy approaches.

## Introduction

Merkel cell carcinoma (MCC) is a rare neuroendocrine, cutaneous malignancy with an incidence rate of 0.13 per 1,00,000 residents in Europe between 1995 and 2002 (1). Therapy consists of surgery only (if N0), surgery followed in most cases by adjuvant radiotherapy (RT) or, more recently, by novel approaches, including immune-checkpoint inhibitors (ICI) in metastatic disease (2–4). The 5-year MCC-specific mortality rate is up to 46% (5, 6). MCC tumorigenesis is linked to the presence of clonally integrated Merkel cell polyomavirus (MCPyV) in up to 80% of the cases, or mutagenesis from ultraviolet light (UV) exposure for MCPyV-negative tumors, as well as advanced age and immunosuppression (3, 7). MCPyV integrates into the host cells genome and persistent expression of MCPyV T antigens is required for MCC tumor cell survival (8). Immunosuppression due to, e.g., organ transplantation or chronic lymphatic leukemia significantly increases the risk for MCC, thus indicating a pivotal role of the host immune system in tumorigenesis (7).

Although it has been reported that patients with high intratumoral CD8+ and CD4+ lymphocyte infiltration show better clinical outcome, including complete spontaneous tumor regression (9–11), the majority of MCC tumors progress despite the presence of T-cells priming MCPyV capsid proteins and oncoproteins. MCC seems to be capable of escaping immune response via down-regulation of major histocompatibility complex class I (MHC-I), Toll-like Receptor 9 (TLR9), and prevention of NF-kB translocation into the nucleus (8, 12). Upregulation of programmed death ligand 1 (PD-L1) expression in response to interferon-γ, released by CD8+ TILs as an adaptive immune-resistance mechanism, can suppress local effector T-cell function. ICI against the PD-1/PD-L1 axis have shown promising results in the treatment of metastatic MCC, and recently resulted in the approval of Avelumab (anti-PD-L1) by the Food and Drug Administration (FDA) (4).

In this study we aimed to correlate MCPyV quantitative viral load, CD8+ tumor infiltrating lymphocytes (TILs), and PD-L1 expression with clinicopathological characteristics and overall survival (OS) in patients with MCC.

## Patients and Methods

### Patients and Treatment

We retrospectively analyzed 49 patients treated for histologically-proven MCC between June 2000 and September 2017 at the Departments of Dermatology and/or Radiotherapy of the University of Frankfurt, Germany. All patients underwent physical examination and complete tumor excision. In case of >cT1 or cN1 cM0, a sentinel lymph node biopsy (SLNB) was performed, followed, in case of positive SLNB, by a regional lymph node dissection and in most cases by adjuvant RT. Depending on tumor site and volume, RT was administered using 3D-conformal or intensity-modulated radiotherapy (IMRT, since 2010) utilizing photon or electron beams and energies ≥6 MV. RT-doses ranged between 20.0 and 70.0 Gray (Gy, median: 60.0 Gy). All patients provided informed consent for sample and clinical data collection. All procedures performed in this study followed approval of our institutional ethics committee (No. 4/09 UCT-03-2017) and were in accordance with the standards of the 1964 Helsinki declaration and its later amendments.

### Immunohistochemistry

Formalin fixed paraffin embedded (FFPE) tumor samples derived from the Dr. Senckenberg Institute of Pathology, and the Department of Dermatology, University of Frankfurt, were subjected to an automatic staining procedure with standardized DAKO EnVision™ FLEX Peroxidase Blocking reagent (K8000, DAKO, Hamburg, Germany) on a DAKO Autostainer Link 48 (DAKO). Antigen retrieval was performed by treatment of the sections using an Epitope Retrieval Solution (Trilog, Cell Marque, Rocklin, CA) for 20 min. Slides were stained with the primary antibodies for either CD8 (1:100, clone C8/144B; Dako M7103) or PD-L1 (1:50, clone E1L3N(R); Cell Signaling Technology) for 120 min at room temperature. Next, dextran polymer conjugated horseradish peroxidase and 3,3′-diamino-benzidine (DAB) chromogen were used for visualization and hematoxylin solution (Gill 3, Sigma Aldrich, Munich, Germany) for counterstaining. Blinded samples were evaluated by two investigators (J.V. and P.B.) without knowledge of the clinicopathologic and clinical data as described before (13, 14). In cases of discrepancy, a final decision was made after additional examination of the specimens. The expression of CD8+ TILs was scored semi-quantitatively via measurement of cell density. Scoring was as follows: for the intra-epithelial, invasive front and stromal compartments: (i) no, or sporadic cells; (ii) moderate numbers of cells; (iii) abundant occurrence of cells; and (iv) highly abundant occurrence of cells. The total score was calculated by adding the separate scores from all three compartments (range, 3–12). The median score was used as cut-off to classify patients into two groups: low (< median) or high (≥median) CD8+ infiltration. PD-L1 tumor expression as evaluated for each sample in different representative fields and expression in >1% of the tumor cells were considered positive as reported before (15).

### MCPyV Detection and DNA Load Determination

Determination of MCPyV DNA load and MCPyV integration status were performed on five 10 μm FFPE sections using a LightCycler 480 Real Time PCR System (Roche, Mannheim, Germany) as described previously (16, 17). Briefly, viral DNA load was determined using MCPyV-specific LT3-primers and a locked nucleic acid probe binding to the N-terminal part of the large T-antigen gene (18). MCPyV DNA load was expressed as MCPyV DNA copies per betaglobin-gene copy (17). The integration status of the MCPyV DNA into the cellular host genome was assessed with a real-time PCR-based MCPyV T-antigen gene C-terminus deletion assay as described before (16). For statistical analysis, a non-detectable viral DNA load was defined as 0 and the median was calculated for the entire cohort (*n* = 48).

### Statistical Analysis

The association of MCPyV, CD8+ infiltration and PD-L1 expression with clinicopathological characteristics was assessed using Pearson's Chi-squared test for categorical variables and Mann-Whitney U test for continuous variables. The clinical outcome measure was overall survival (OS) as defined from the time-point of histologically confirmed diagnosis of MCC to death from any cause. Differences in OS between groups were plotted using the Kaplan–Meier method and assessed using the Log-rank test (Mantel-Cox; SPSS 25). A *p* < 0.05 was considered as significant.

## Results

### Patients and Tumor Characteristics

From a total of 49 patients, 25 (51.0%) were female. Median age at diagnosis was 74 (range, 42–100) years. The head was the main tumor site (45.5%), followed by arms (34.0%), and body trunk (20.5%). A total of 54.5% of the patients had positive lymph nodes, and 44.9% received adjuvant RT. Concerning the MCPyV-DNA status, 1 MCC was not evaluable due to low cellularity (betaglobin-gene copy number < 10), 38 of the remaining 48 biopsies were MCPyV-DNA positive (79.2%), and 10 MCC (20.8%) were MCPyV-negative. The median viral DNA load for the entire cohort (*n* = 48) was 0.745 (interquartile range 0.007–4.448; mean 7.072; range 0.000–157.007). Integrated, C-terminally deleted MCPyV-DNA was found in 22.9% of all patients (11/48), episomal or full-length integrated MCPyV-DNA in 33.3% of all patients (16/48), and in 22.9% of the entire cohort (11/48) the integration status could not be evaluated or was negative (20,8%, 10/48). Patient characteristics are given in Table 1.

**Table 1:** Patients characteristics.

**Clinical characteristics**	***n* (%)**
**Total (*****n*** **= 49)**	
**GENDER**	
Male	24 (49.0)
Female	25 (51.0)
Age, median (range)	74 (42–100)
**TUMOR LOCALIZATION**	
Head	20 (45.5)
Arm	15 (34.0)
Body trunk	9 (20.5)
Missing values	5
**cN-CATEGORY**	
cN0	15 (45.5)
cN+	18 (54.5)
Missing values	16
**CD8 SCORE**^‡^	
<median	25 (51.0)
≥median	24 (49.0)
PD-L1*	
≤1%	21 (42.9)
>1%	28 (57.1)
**MCPyV DNA STATUS**	
Positive	38 (79.2)
Negative	10 (20.8)
Not assessable	1
**VIRAL INTEGRATION STATUS**	
Integrated,C-terminally deleted	11 (22.9%)
Episomal or full-length integrated	16 (33.3%)
Integration status not assessable	11 (22.9%)
MCPyV-negative	10 (20.8%)
Missing values	1
**RADIOTHERAPY**	
Yes	22 (44.9)
No	27 (55.1)

*MCPyV, Merkel Cell Polyomavirus*.

* *% PD-L1+ tumor cells*.

‡ *CD8+ tumor infiltration*.

### Clinicopathological Characteristics and Their Association With MCPyV Status, CD8 Infiltration, and PD-L1 Expression

**Figure 1:**
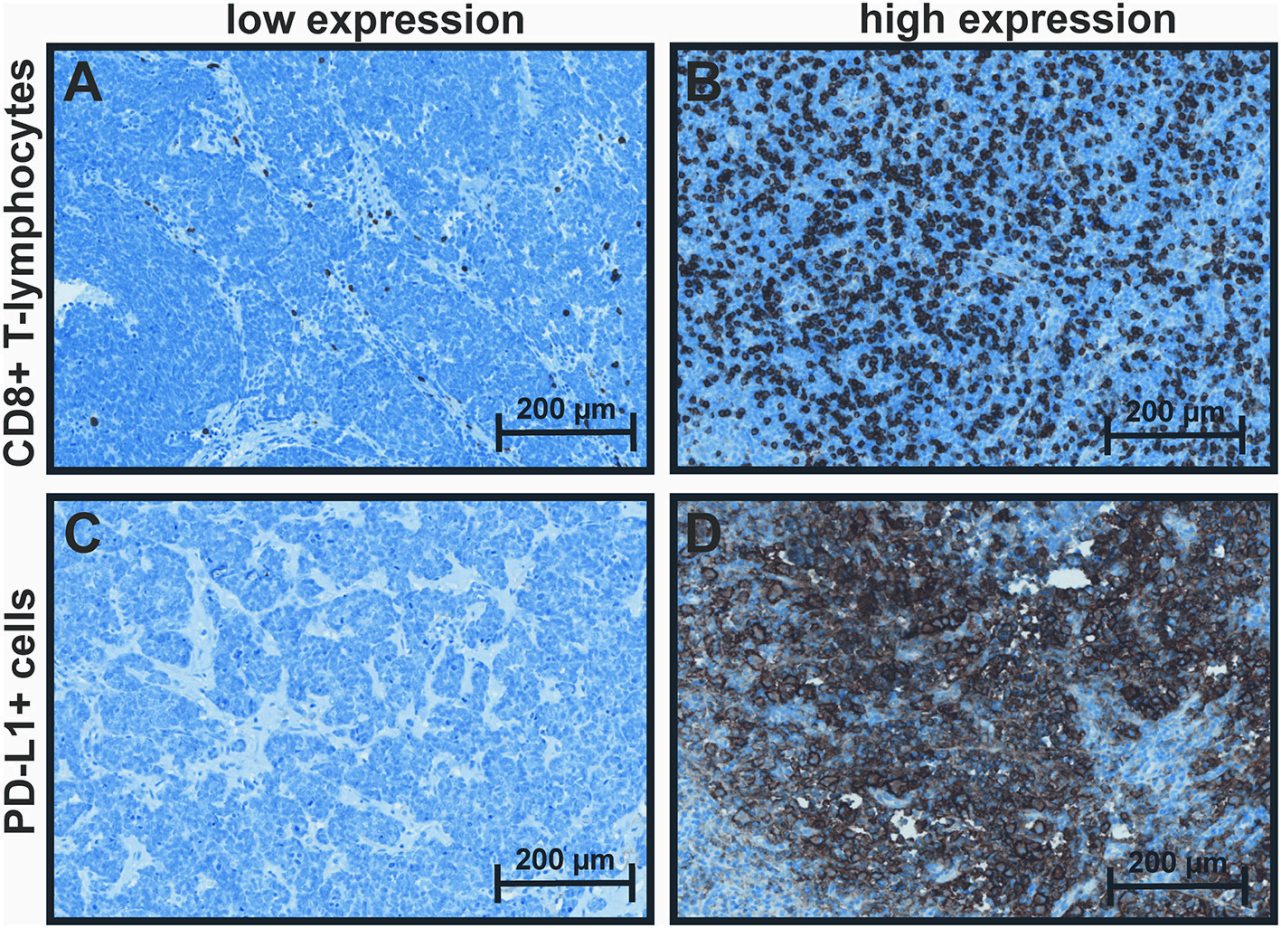
Immunohistochemical staining of CD8 and PD-L1. **(A)** CD8: low: < median score of 5, **(B)** high: ≥median score of 5 (range 3–12); **(C)** PD-L1: ≤ 1% positive tumor cells (low) and **(D)** >1% positive tumor cells (high).

For CD8+ TILs, the median score was used as cut-off to dichotomize between low and high infiltration, whereas PD-L1+ expression in >1% of the tumor cells was considered positive (Figure 1). Tumor localization (head/arms vs. trunk) positively correlated with PD-L1 status (p = 0.011, Table 2) and combined CD8/PD-L1 expression (p = 0.038, Supplementary Table 2). Overall CD8+ infiltration was inversely associated with N-stage (p = 0.048, Table 2). A high stromal CD8+ infiltration was associated with PD-L1 positivity (p = 0.010) and N-stage (p = 0.037, Table 3). Further, a positive MCPyV status and high viral DNA load were associated with female gender (p = 0.042 and 0.021, respectively) (Table 2 and Supplementary Table 1).

**Table 2:** Clinicopathological characteristics and their association with MCPyV status and immune microenvironment.

**Clinico-pathological characteristics**	**MCPyV status**, ***n*** **(%)**		***p***	**CD8** ***n*** **(%)**		***p***	PD-L1*, ***n*** **(%)**		***p***
	**Negative^#^**	**Positive**		**<Median**	**≥Median**		**≤1%**	**>1%**
**N-STAGE (*****n*** **= 33)**									
Negative	7 (46.7)	8 (53.3)		4 (26.7)	11 (73.3)		7 (46.7)	8 (53.3)
Positive	7 (38.9)	11 (61.1)	0.653	11 (61.1)	7 (38.9)	**0.048**	7 (38.9)	11 (61.1)	0.653
**TUMOR LOCALIZATION (*****n*** **= 45)**									
Head or arms	16 (45.7)	19 (54.3)		20 (57.1)	15 (42.9)		16 (45.7)	19 (54.3)
Other	3 (33.3)	6 (66.7)	0.504	3 (22.2)	7 (77.8)	0.062	0 (0)	9 (100.0)	**0.011**
PD-L1* (***n*** **= 49)**									
≤ 1%	9 (45.0)	11 (55.0)		14 (66.7)	7 (33.3)			
>1%	12 (42.9)	16 (57.1)	0.883	11 (39.3)	17 (60.7)	0.058		
**CD8**^‡^**(*****n*** **= 49)**									
< median	11 (45.8)	13 (54.2)					14 (56.0)	11 (44.0)
≥median	10 (41.7)	14 (58.3)	0.771				7 (29.2)	17 (70.8)	0.058
**MCPyV STATUS (*****n*** **= 48)**									
Negative^#^				7 (70.0)	3 (30.0)		4 (40.0)	6 (60.0)
Positive				17 (44.7)	21 (55.3)	0.155	16 (42.1)	22 (57.9)	0.503
**GENDER (*****n*** **= 49)**									
Male	14 (58.3)	10 (41.7)		12 (50.0)	12 (50.0)		9 (37.5)	15 (62.5)
Female	7 (29.2)	17 (70.8)	**0.042**	13 (52.0)	12 (48.0)	0.889	12 (48.0)	13 (52.0)	0.458

*MCPyV, Merkel Cell Polyomavirus; PD-L1,Programmed cell death ligand 1*.

* *% PD-L1+ tumor cells*.

‡ *CD8+ tumor infiltration, overall score*.

# *defined as negative or not assessable †p-values according to Pearson's Chi-squared test and calculated after exclusion of missing values*.

Significant results have been marked with bold.

**Table 3:** PD-L1 and N-stage and their association with stromal CD8 infiltration.

**Clinico-pathological characteristics**	**Stromal CD8+ infiltration, n (%)**		**p**^†^
	**<Median**	**≥Median**
PD-L1* **(n = 49)**			
≤ 1%	12 (57.1)	9 (42.9)
>1%	6 (21.4)	22 (78.6)	**0.010**
**N-STAGE (n = 33)**			
Negative	3 (20.0)	12 (80.0)
Positive	10 (55.6)	8 (44.4)	**0.037**

*PD-L1,Programmed cell death ligand 1*.

* *% PD-L1+ tumor cells*.

† p-values according to Pearson's Chi-squared test and calculated after exclusion of missing values. Significant results have been marked with bold.

### Overall Survival and Correlation With MCPyV DNA Load, CD8, and PD-L1

**Figure 2:**
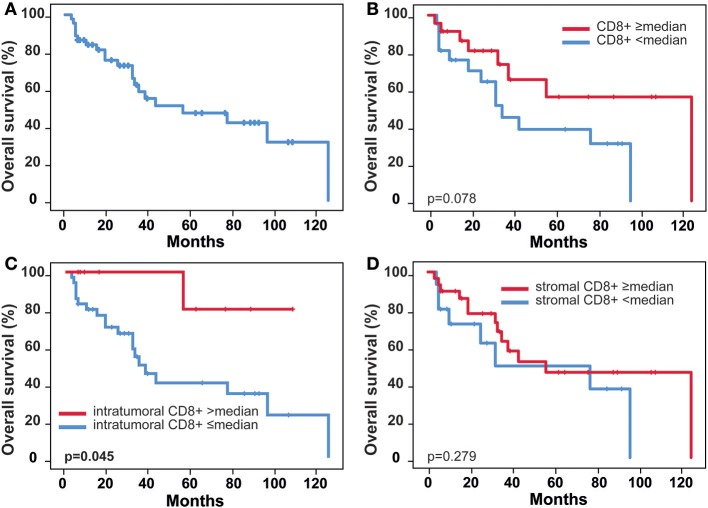
Overall survival stratified by CD8 immune infiltration. **(A)** Overall survival, **(B)** Overall survival stratified by CD8 median score, **(C)** Overall survival stratified by CD8 intratumoral median score, **(D)** Overall survival stratified by CD8 stromal median score; p-values according to log-rank test (Mantel Cox).

**Figure 3:**
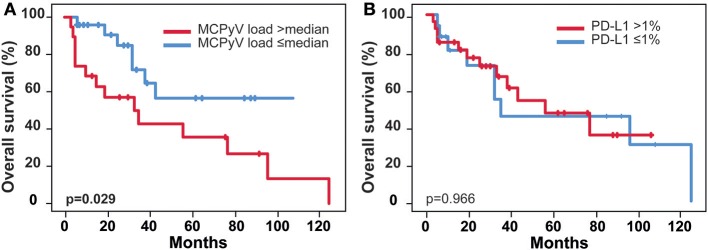
Patients outcome and correlation with MCPyV DNA load and PD-L1. **(A)** Overall survival stratified by MCPyV DNA median load (n = 48), **(B)** Overall survival stratified by PD-L1 status; MCPyV, Merkel Cell Polyomavirus; p-values according to log-rank test (Mantel Cox).

One-, three-, and five-year OS rates were 83.8, 58.6, and 47.1%, respectively (Figure 2). Cumulative (p = 0.078) and stromal (p = 0.279) expression of CD8+ TILs were not associated with OS, whereas elevated levels of intratumoral CD8+ cells correlated significantly with superior OS for the entire cohort (p = 0.045, Figure 2). High levels of DNA viral load (>median) were significantly related to a worse OS (p = 0.029, Figure 3). The association remained significant after exclusion of cases that lack detectable viral DNA (p = 0.034 for n = 38, Supplementary Figure 1). PD-L1-positivity did not correlate with OS (p = 0.966).

## Discussion

MCC is an aggressive disease with various options of the malignant cells to avoid immune response. Accumulating evidence indicates a direct association of higher “immunogenicity” and response to RT in MCC (19), and other virus-associated malignancies, including HPV-16/18 induced oropharyngeal and anal carcinoma (13, 20, 21). A recent investigation in 805 patients with MCC indicated a significantly impaired efficacy of RT in terms of local tumor control and recurrence-free survival for patients with immunosuppression (22). Further understanding of tumor driving mechanisms may lead to new strategies facing this rare tumor entity.

In the present study, we quantitatively evaluated the prevalence, viral load, and genomic integration into the host DNA of MCPyV in a cohort of MCC-patients and correlated these parameters with OS, PD-L1 status, and CD8+ lymphocyte infiltration. To the best of our knowledge, this is the first study investigating the relationship between MCPyV viral load and survival (9, 23). Vandeven et al. could demonstrate that MCC of unknown primary (MCUP) was associated with higher levels of MCPyV-antibodies and higher mutational load, as surrogate parameters for immunogenicity, and correlated with improved survival when compared to patients with identified primary tumors (24). Additionally, other authors have reported a positive correlation of a high antibody titer with MCPyV status and OS for classical MCC (18, 25, 26). These data provide a strong rationale for a virus-triggered effective immune-activation as a pivotal mechanism underlying tumor elimination.

Intriguingly, a high viral load correlated with worse OS in our cohort while tumors with a lower load or lack of viral DNA displayed increased OS. MCPyV negative tumors are mainly considered to be induced by ultraviolet radiation and present a high mutational burden in general and more specifically high incidence of p53 (75%) and Rb mutations (67%) (27, 28). Emerging evidence shows a clear association of mutational load and prognosis for almost any malignancy, a phenomenon associated with the increased immunogenicity of such tumors (29). The percentage of non-virally induced tumors in our cohort is in accordance with the literature (28, 30). These extensively—mutated cases could have an even better outcome compared to MCPyV-driven tumors, such biasing the survival-analysis. Moreover, a less favorable outcome for MCPyV negative tumors has been reported before (25, 31). However, the significance for the correlation of high viral load and OS still remained after exclusion of cases without any detectable viral DNA. A possible reason for the impaired survival of patients with high viral-load is a missing or ineffective immune response due to immunosuppression or various cancer- and microenvironment-associated mechanisms, including alteration of regulatory T cell function and activation of the PD-1/PD-L1 axis (32, 33). Notably, similar findings have been reported for Epstein-Barr-Virus (EBV) associated nasopharyngeal cancer, where a high EBV-DNA load in the plasma correlated with an impaired outcome (34, 35).

Until the advent of ICI, chemotherapy was standard of care in the treatment of advanced MCC. First-line platinum-based chemotherapy combined with Etoposide showed overall response rates (ORR) of 31–55% with shorter progression-free survival than those recently reported for anti-PD-1/PD-L1 ICI (3). In a recent phase 2 trial the anti-PD-L1 antibody Avelumab was applied to 88 patients with stage IV MCC that had progressed after chemotherapy. Objective response was reached in 32% of the patients indicating superiority of novel immune-modulating therapies (2). These findings resulted in the first approval of a checkpoint inhibitor in MCC (4). Other studies investigating anti-PD-1 antibodies Nivolumab (+/- prior chemotherapy, recruiting) and Pembrolizumab (no prior chemotherapy) reported ORR of 68 and 56%, respectively (36, 37). In our cohort, PD-L1 status, however, was not associated with altered outcome, suggesting that this marker may be predictive for response to targeted therapy but not prognostic.

Regarding infiltration with CD8-positive cytotoxic lymphocytes, we identified a significant correlation between intratumoral CD8+ infiltration and OS, and a significant inverse correlation with nodal-stage (a widely accepted negative prognosticator for MCC). Notably, N+ disease in our cohort occurred in 54.5% of the cases while literature reports on 37% (7), a fact attributed to selection bias, as many of the patients included here were referred to the department of radiotherapy. In a larger study by Paulson et al., both clinical stage and CD8-infiltration were of prognostic relevance (10). More recent analyses of larger numbers of samples seem to confirm these assumptions (9, 38, 39) and the same was true when the specificity of T cells for MCPyV was taken to account (40). Interestingly, we did not observe any significant correlation between total tumor CD8-infiltration, PD-L1 expression, and viral load, indicating that mechanisms other than viral infection (e.g., ultraviolet radiation-induced mutations) may contribute to immune response. On the contrary, stromal infiltration with CD8+ TILs significantly correlated with both PD-L1 and MCPyV. This argues for a locally restricted, viral antigene-driven immune response that failed to control the tumor in a PD-L1 dependent manner, that could be potentially reversed by ICI (41).

With respect to the correlation of MCPyV status/viral DNA load with clinical and epidemiological parameters, the most important finding in the present cohort was a significant correlation with female gender although the limited number of patients in our study does not allow definite conclusions yet. In line with that, the higher prevalence of MCPyV in female patients has been reported before, but a possible association with tumor site remains controversial (18, 42, 43). There is no molecular explanation readily available for the increased prevalence in women. A putative reason, however, may be the observation that tumors in females were diagnosed more frequently in older patients (median age females 77.0 years vs. median age males 70.5). In line with that, Álvarez-Argüelles et al. recently speculated that there may an immunosuppressive component due to age contributing to the sex effect in MCPyV detection demonstrated in their analyses and in our study (44). Unfortunately we could not prove an association of age and viral load in our data. Another possible explanation could be a higher UV-exposure as casual factor in the male population. Yet there exist no data to undermine this speculation, although a viral etiology has been associated with female sex by many authors (18, 42, 43). Interestingly, male sex, and advanced age were associated with worse prognosis in the literature (7).

We acknowledge that the retrospective evaluation and the small number of patients is a limitation of our study. A potential calculation bias cannot be excluded. However, this is the first study quantitatively assessing and correlating the MCPyV viral load to clinical parameters that warrant validation in larger, independent cohorts with long-term follow-up.

## Conclusion

These data provide important insight on the crucial role of MCPyV DNA load and TILs, in the context of PD-L1, in patients with MCC. We consider our findings on a correlation of PDL-1 with tumor localization and CD8+ Tils and a prognostic relevance of intratumoral CD8+ T cell infiltration to be in favor of a future checkpoint immunotherapy in MCC. Moreover, there is growing pre-clinical and clinical evidence on an additional improvement of the effects of checkpoint-inhibition by synergistic effects of radiation therapy (45, 46). Consequently, future clinical studies should aim to explore the effect of PD-1/PD-L1 immune-checkpoint inhibitors in combination with existing radiotherapy approaches.

## Ethics Statement

This study was carried out in accordance with the recommendations of the local ethics committee (Frankfurt University No. 4/09 UCT-03-2017) with written informed consent from all subjects. All subjects gave written informed consent in accordance with the Declaration of Helsinki. The protocol was approved by the Frankfurt university ethics committee.

## Author Contributions

JG, RW, FR, and PB conceived the idea. JG, RW, CR, and MM provided patient data and material. RW, MM, UW, and SS contributed to the sample-preparations. RW, UW, and SS carried out the laboratory analyses. JG, RW, FR, and PB performed microscopy. JG, DM, and PB performed the statistics. JG, DM, FR, and PB analyzed and interpreted the data. FR, UW, EF, and CR were involved in the planning and supervising. JG, DM, FR, and EF drafted the manuscript and designed the figures. JG, EF, CR, FR, and PB wrote the manuscript, with contributions from the other authors. All authors read and approved the final manuscript.

### Conflict of Interest Statement

The authors declare that the research was conducted in the absence of any commercial or financial relationships that could be construed as a potential conflict of interest.

## Supplementary Material

Supplementary Figure 1 Patients outcome and correlation with MCPyV DNA load. Overall survival stratified by MCPyV DNA median load restricted to patients with any detectable viral DNA independent from integration status (*n* = 38); *p*-value according to log-rank test (Mantel Cox).

Supplementary Table 1 Association of MCPyV and gender.

Supplementary Table 2 Clinicopathological characteristics and their association with combined CD8/PD-L1 expression.
