# Supplementary figures and images for: Merkel Cell Polyoma Viral Load and Intratumoral CD8+ Lymphocyte Infiltration Predict Overall Survival in Patients With Merkel Cell Carcinoma
Source: Front Oncol. 2019 Jan 24;9:20. doi: 10.3389/fonc.2019.00020 (PMC6354572; doi:10.3389/fonc.2019.00020)

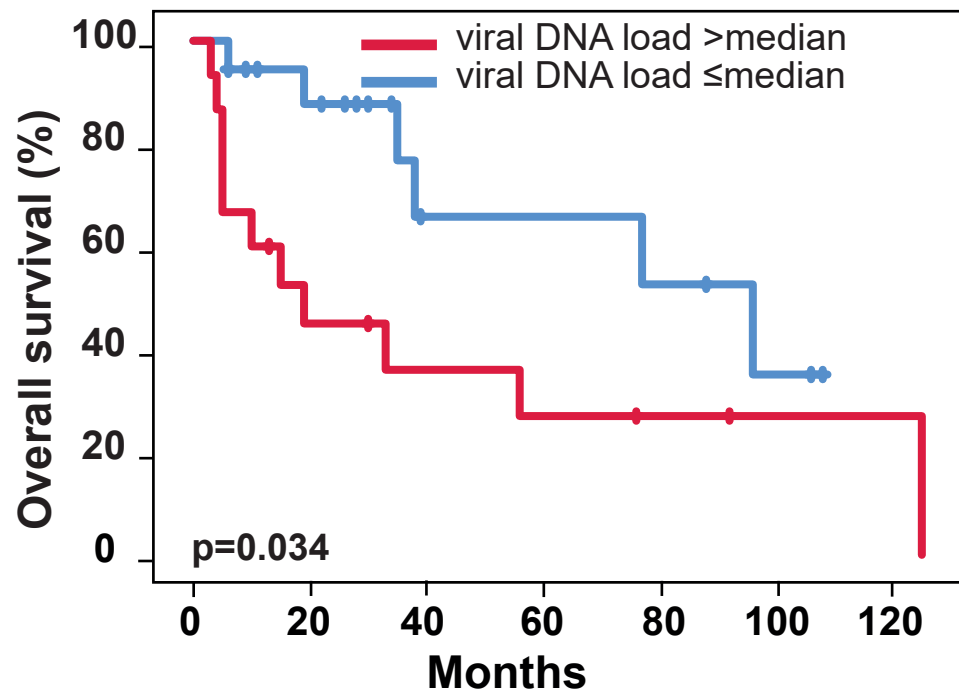

Supplement: Supplementary Figure 1 — Patients outcome and correlation with MCPyV DNA load. Overall survival stratified by MCPyV DNA median load restricted to patients with any detectable viral DNA independent from integration status (n = 38); p-value according to log-rank test (Mantel Cox). [file Image_1.pdf]
