# Supplementary material for: Merkel Cell Polyoma Viral Load and Intratumoral CD8+ Lymphocyte Infiltration Predict Overall Survival in Patients With Merkel Cell Carcinoma
Source: Front Oncol. 2019 Jan 24;9:20. doi: 10.3389/fonc.2019.00020 (PMC6354572; doi:10.3389/fonc.2019.00020)
Supplement: Supplementary Table 1 — Association of MCPyV and gender. [file Table_1.docx]

**Supplementary Table 1.** Association of MCPyV and gender

| MCPyV characteristics | Gender, n (%) | | p^†^ |
| --- | --- | --- | --- |
| Total n=48 | male | female |  |
| Viral integration status |  |  |  |
| integrated, C-terminally deleted | 7 (63.6) | 4 (36.4) |  |
| episomal or full-length integrated | 3 (18.8) | 13 (81.3) |  |
| Integration status not assessable, MCPyV-positive | 6 (54.5) | 5 (45.5) |  |
| MCPyV-negative | 8 (80.0) | 2 (20.0) | **0.013** |
| Viral DNA load* |  |  |  |
| ≤median | 16 (66.7) | 8 (33.3) |  |
| >median | 8 (33.3) | 16 (66.7) | **0.021** |

Abbreviation: MCPyV - Merkel Cell Polyomavirus; *MCPyV DNA median load restricted to patients with any detectable viral DNA independent from integration status (n=38); ^†^p-values according to Pearson’s Chi-squared test for categorial variables and Mann-Whitney-U test for continuous variables. Significant results have been marked with bold.
