# Supplementary material for: Merkel Cell Polyoma Viral Load and Intratumoral CD8+ Lymphocyte Infiltration Predict Overall Survival in Patients With Merkel Cell Carcinoma
Source: Front Oncol. 2019 Jan 24;9:20. doi: 10.3389/fonc.2019.00020 (PMC6354572; doi:10.3389/fonc.2019.00020)
Supplement: Supplementary Table 2 — Clinicopathological characteristics and their association with combined CD8/PD-L1 expression. [file Table_2.docx]

**Supplementary table 2**. Clinicopathological characteristics and their association with combined CD8/PD-L1 expression

| Clinico-pathological characteristics | Combined CD8^‡^/PD-L1* expression, n (%) | | | | p^†^ |
| --- | --- | --- | --- | --- | --- |
|  | CD8 high  PD-L1 high | CD8 low  PD-L1 high | CD8 high  PD-L1 low | CD8 low  PD-L1 low |  |
| Tumor localization | |  |  |  |  |
| Head or arm | 10 (28.6) | 10 (28.6) | 4 (11.4) | 11 (31.4) |  |
| other | 7 (77.8) | 2 (22.2) | 0 (0) | 0 (0) | **0.038** |
| MCPyV status | |  |  |  |  |
| negative | 2 (20.0) | 4 (40.0) | 1 (10.0) | 3 (30.0) |  |
| positive | 15 (39.5) | 8 (21.1) | 5 (13.2) | 10 (26.3) | 0.554 |
| MCPyV viral load | |  |  |  |  |
| ≤median | 11 (44.0) | 5 (20.0) | 4 (16.0) | 4 (16.7) |  |
| >median | 6 (25.0) | 7 (29.2) | 2 (8.3) | 9 (37.5) | 0.222 |
| Gender |  |  |  |  |  |
| male | 7 (29.2) | 8 (33.3) | 5 (20.8) | 4 (16.7) |  |
| female | 10 (40.0) | 4 (16.0) | 1 (4.0) | 10 (40.0) | 0.069 |

Abbreviations: MCPyV - Merkel Cell Polyomavirus, PD-L1 – Programmed cell death ligand 1; *PD-L1+ cells high: <1% of all cells positive, low: ≥1% of all cells positive; ‡CD8+ tumor infiltration high: ≥median, low: <median. Significant results have been marked with bold.
